# Long-range two-photon scattering spectroscopy ruler for screening prostate cancer cells

**DOI:** 10.1039/c4sc03843f

**Published:** 2015-02-03

**Authors:** Sudarson Sekhar Sinha, Dilip K. Paul, Rajashekhar Kanchanapally, Avijit Pramanik, Suhash Reddy Chavva, Bhanu Priya Viraka Nellore, Stacy J. Jones, Paresh Chandra Ray

**Affiliations:** a Department of Chemistry and Biochemistry , Jackson State University , Jackson , MS , USA . Email: paresh.c.ray@jsums.edu ; Fax: +1 6019793674

## Abstract

Optical rulers have served as a key tool for scientists from different disciplines to address a wide range of biological activity. Since the optical window of state of the art FRET rulers is limited to a 10 nm distance, developing long range optical rulers is very important to monitor real life biological processes. Driven by this need, the current manuscript reports for the first time the design of long-range two-photon scattering (TPS) spectroscopy rulers using gold nano-antenna separated by a bifunctional rigid double strand DNA molecule, which controls the spectroscopy ruler length. Reported data demonstrate that the TPS spectroscopy ruler's working window is a within a 25 nm distance, which is more than twice that of well recognized FRET optical ruler. A possible mechanism for the two-photon spectroscopy ruler's long range capability have been discussed using angle-resolved TPS measurement and FDTD simulations. Solution-phase experimental data demonstrated that a long-range TPS ruler using A9 aptamer can be used for the screening of prostate-specific membrane antigen (PSMA) (+) prostate cancer cells even at 5 cells per mL level. Reported result with PSMA (–) normal skin HaCaT cells indicate that TPS ruler based assay has the capability to enable distinction from non-targeted cell lines. Ultimately, the long range TPS ruler can be used towards better understanding of chemical and biological processes.

## Abstract 2

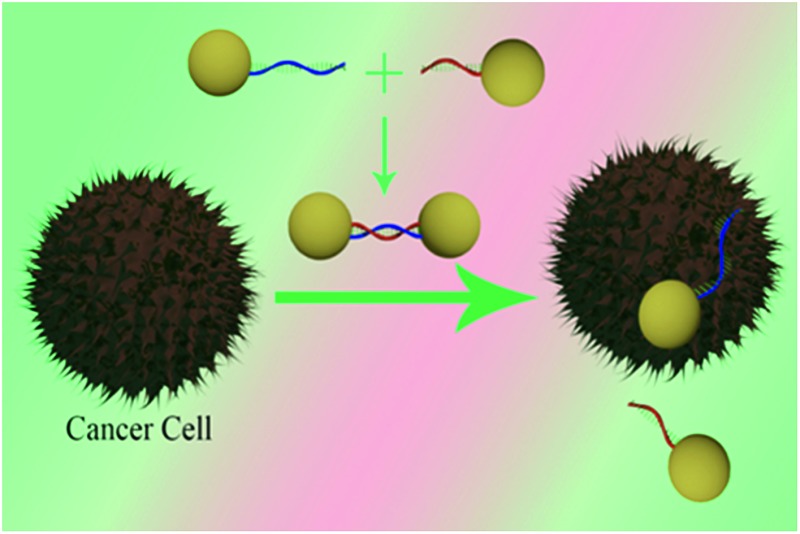
Development of a long-range TPS ruler for the screening of prostate cancer cells with sensitivity of 5 cells per mL level is demonstrated.

## Introduction

**Scheme 1:**
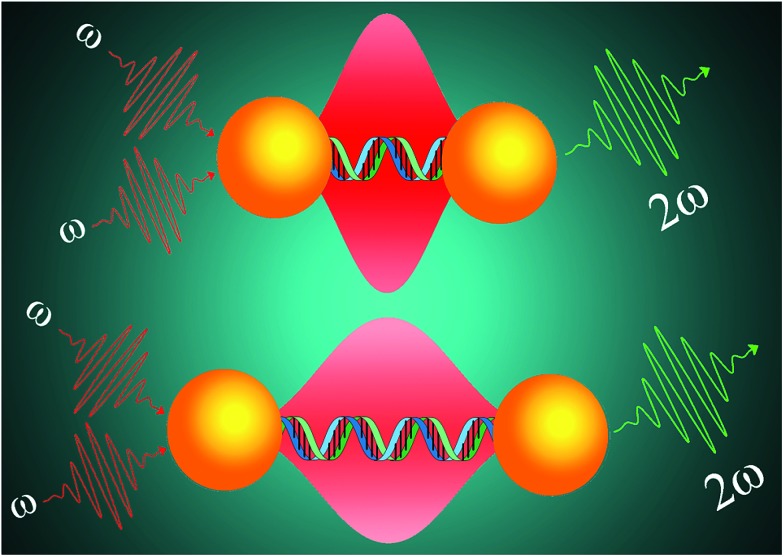
Schematic representation illustrating the design of two-photon scattering spectroscopy ruler using gold nanoparticle and ds-DNA.

Since the introduction of Förster resonance energy transfer (FRET) by Stryer and Haugland in 1967, optical spectroscopy rulers using FRET have become an essential tool for scientists from different disciplines to address a wide range of biological activity.^1–4^ Since the existing FRET spectroscopy ruler distance window is governed by dipole–dipole coupling mechanism, scientists face problems monitoring biological processes that occur at greater than 10 nm scale distance.^1–4^ To overcome the distance limitation, in past few years scientists have been trying to develop nanomaterial based optical rulers.^5–19^ Using this approach, the current article reports for the first time the development of a long range two-photon scattering (TPS) spectroscopy ruler, whose distance window is more than twice that of the 10 nm distance limit, as shown in Scheme 1.

When an intense laser light of frequency *ω* is exposed to a sample, it generates scattered radiation with the frequency 2*ω*, which is known as two-photon scattering or second harmonic (SH) light.^20–27^ It is now well documented that when two plasmonic nanostructures are in close proximity, the electric field in the hot spot is enhanced by several orders of magnitudes.^20–25,28–31^ Since the two-photon scattering is directly proportional to the fourth power of the fundamental field amplitude, theoretically a huge two-photon scattering enhancement is expected due to the formation of a nano-antenna structure.^20–25,28–34^ The above fact motivated us to develop a long-range TPS ruler, where we have used the advantage of the strong plasmonic electric field generated by gold nanoparticle antennas to demonstrate that the distance window for a TPS ruler can be tuned up to 25 nm. Our experimental finding on the distance dependent two-photon scattering are supported by finite difference time domain (FDTD) simulation investigation of the distance-dependent two-photon scattering properties.^21,24–26,30,32–34^ In our calculation, we have included multipolar and finite size effects and these are very important for gold nano-antenna of size close to the wavelength of light.^21,24–26,30^

To understand the possible application of TPS ruler for biological screening, we have demonstrated the detection of prostate-specific membrane antigen (PSMA) (+) prostate cancer cells using our developed TPS ruler in solution phase. PSMA is highly expressed on the surface of prostate cancer, which is the second leading cancer in the male population.^35–37^ PSMA is a prostate cancer-specific biomarker which has been correlated with aggressive disease and used for monitoring of therapy.^35–37^ Since the ability to detect very low levels of PSMA may enable doctors to diagnose men with prostate cancer recurrence, it has the capability to reduce prostate cancer-related mortality and morbidity. During the last decade several articles have shown that two-photon scattering properties can be enhanced (10^6^ to 10^9^) in magnitude by using metal nanoparticle-surfaces, which is comparable to the very large enhancement of surface-enhanced Raman scattering.^22–26,28,37^ Here we show that an anti-PSMA aptamer based TPS ruler can be used for ultrasensitive detection of PSMA (+) LNCaP prostate cancer cells. For this purpose, we have developed a TPS ruler using the partial sequence of A9 aptamer,^35,36^ which has the capability to target clinically relevant LNCaP prostate cancer. A9 aptamer is known to recognize and bind to PSMA in intracellular or cytoplasmic epitope.^35,36^ Our experimental data with PSMA negative normal skin HaCaT cells, indicate that spectroscopy ruler based assay is highly selective and enables their distinction from non-targeted cancer cell lines.

## Results and discussion

**Fig. 1:**
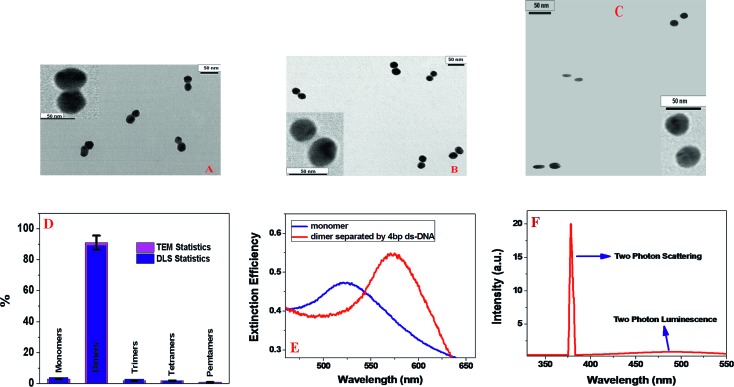
(A) TEM image shows gold nanoantenna, with 3 nm separation between two individual nanoparticles when they are separated by thiolated 4 bp ds-DNA. (B) TEM image shows gold nanoantenna with 8 nm separation between two individual nanoparticles, when they are separated by thiolated 20 bp ds-DNA. (C) TEM image shows that gold nanoantenna with 27 nm separation between two individual nanoparticles when they are separated by thiolated 80 bp ds-DNA. (D) DLS and TEM statistics of gel-purified 20 bp dimers sample. For TEM statistics we have measured an average of 950 particles. Measurement has been performed 6 times and error bars have been determined based on the measurement. (E) Extinction spectra for monomer and dimer, separated by 4 bp ds-DNA. (F) Emission spectrum from gold dimer with 3 nm separation at 760 nm excitation. The spectrum shows a strong SHG peak at 380 nm. It also indicates very weak and broad two photon luminescence with emission maxima at 480 nm.

A long-range two-photon spectroscopy ruler from 2 to 30 nm range was designed using SH-modified ds-DNA as a coupling agent. We have followed an established technique as reported by Alivisatos *et al*.^12,13^ Initially, thiolated ss-DNA molecules (HS–(CH_2_)_6_–oligo) were conjugated to gold nanoparticles, followed by a passivation layer of thiolated polyethylene glycol. After that, hybridization was performed using a complementary sequence (oligo–(CH_2_)_3_–SH) attached gold nanoparticle in 10 mM PBS solution containing 0.3 M NaCl. To minimize multiple tether formation during hybridization of the gold–ssDNA conjugates, the ssDNA particle ratio was limited to 25 : 1, as reported by Alivisatos *et al.* ^12^ In the next step, dimers were separated from unreacted monomers and larger assemblies by gel-electrophoresis as reported by Alivisatos *et al.* ^12^ Finally, dimers were isolated from the gel by electroelution. After that, the dimer structures were confirmed using dynamic light scattering (DLS) and transmission electron microscopic (TEM) techniques. Since it is known that TEM grid preparation can increase aggregation,^33,34^ to confirm that the isolated assemblies were dimers, we have used dynamic light scattering (DLS) measurement and compared the data with transmission electron microscopic technique (TEM), as shown in Fig. 1D and Table 1. As shown in Fig. 1D, our experimentally measured DLS and TEM statistics data indicate that the gel-purified 20 bp dimer sample is highly enriched in dimers (∼90%). Our data show that the monomers and higher agglomerates are very few. TEM statistics showed that there are an average of 950 particles. Measurement has been performed 6 times and the average values have been reported in this manuscript.

**Table 1:** Spectroscopy ruler distance measured by DLS and estimated using rigid-rod approximation

Ruler system description	Ruler distance using DLS/nm	Estimated ruler distance/nm
Dimer separated by 4 bp ds-DNA	3.0 ± 0.2	2.9
Dimer separated by 10 bp ds-DNA	5.0 ± 0.3	4.8
Dimer separated by 20 bp ds-DNA	9.0 ± 0.4	8.0
Dimer separated by 30 bp ds-DNA	12.0 ± 1.0	11.2
Dimer separated by 40 bp ds-DNA	16.0 ± 1.4	14.4
Dimer separated by 60 bp ds-DNA	22.0 ± 2.0	20.8
Dimer separated by 80 bp ds-DNA	26.0 ± 3.0	27.2

To design TPS rulers in the 2 to 30 nm range, we have used ds-DNA spacers of 4, 10, 20, 30, 40, 60 and 80 base pairs. In the design of spectroscopy rulers in the window from 3 to 30 nm Clegg *et al.* ^18^ have reported that duplex DNA lengths of less than 100 base pairs can be assumed as linear chains by a rigid-rod approximation. As a result, in our design we have estimated the “long range spectroscopy ruler” distance by taking into account the size of the 0.32 nm for each base pair, and total 1.6 nm for Au–S–(CH_2_)_*n*_–DNA base distance in both sides of base pair. To find out the validity of our assumption of the rigid-rod approximation, we have employed dynamic light scattering (DLS) measurement using a Malvern Zetasizer Nano instrument.

As shown in Table 1, experimental DLS data indicate that the gold nanoparticles have an average size of ∼25 ± 2 nm, which also can be seen clearly from the TEM data, as shown in Fig. 1. On the other hand, in nano-antenna, when two gold nanoparticles are separated by 20 base pairs (bp) dsDNA, the diameter changes to ∼59 ± 2 nm, which also matches quite well with the TEM data as shown in Fig. 1B. Similarly, as shown in Table 1, the end-to-end distance measured between gold nanoparticles separated by ds-DNAs of different lengths, and the DLS measurement data are quite close to our estimated distances. Measurement has been performed 6 times and average values have been reported in this manuscript.

Fig. 1E and Table 2 report how the plasmon band for the gold nanoantenna varies with the separation distance. Our reported data indicate that with decreasing interparticle gap, a strong red-shift along with the increase in extinction efficiency are observed. When the gap reaches around 22 nm, the maxima of plasmon band remain unchanged from the monomer absorption band, which is at 528 nm. Observed distance dependent plasmon shift is mainly due to the fact that the magnitude of the plasmon shift for gold nanoantenna is highly dependent on the strength of the interparticle coupling, which in turn, depends on the proximity of the individual nanoparticle.

**Table 2:** Experimental and theoretical plasmon resonance (PR) maxima for gold nano-antenna with different monomer separation distance

Ruler system description	*λ*_PR_/nm (expt)	*λ*_PR_/nm (theory)
Dimer separated by 4 bp ds-DNA	580	590
Dimer separated by 10 bp ds-DNA	550	560
Dimer separated by 20 bp ds-DNA	540	545
Dimer separated by 30 bp ds-DNA	535	536
Dimer separated by 40 bp ds-DNA	532	534
Dimer separated by 60 bp ds-DNA	530	530
Dimer separated by 80 bp ds-DNA	528	528

Two-photon scattering intensity from the monomer and nano-antenna were measured in solution using the hyper-Rayleigh scattering (HRS) technique.^22,23,27,28,30–34^ To measure the TPS intensity, a femtosecond Ti:sapphire laser beam with excitation wavelength of 760 nm was used. Details of the HRS experimental procedure we use in our laboratory have been reported before.^22,23,30^ TEM and DLS experiment performed before and after 5 min of laser exposure indicated no photothermal damage during HRS data collection time and as a result, the number of dimers remained constant. The two-photon scattering light was separated from its single-photon scattering counterpart using a 3 nm bandwidth interference filter and a monochromator. For angle-resolved two-photon scattering intensity measurement, the fundamental input beam at 760 nm light was linearly polarized, and a rotating half-wave plate was used to vary the input angle of polarization. The two-photon scattering intensity from gold nano-antenna can be expressed as:^22,23,27,28,30–34^

**Fig. 2:**
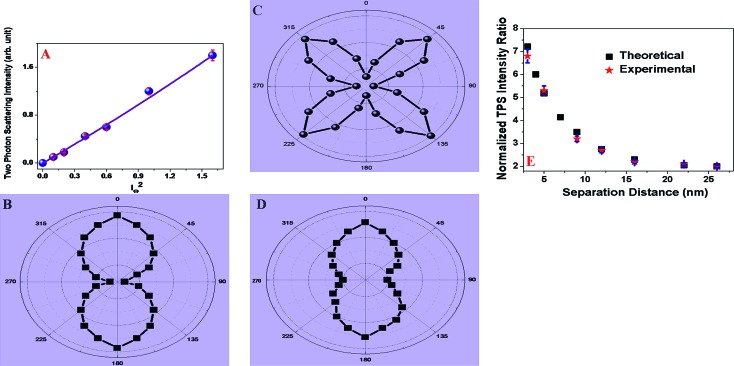
(A) Plot shows how the TPS intensity from gold nano-antenna with 3 nm separation varies with the 760 nm incident light power (W cm^–2^). Observed linear relationship between the square of the powers of the fundamental incident light implies that the signal is indeed due to a two-photon process. (B–E) TPS intensity was recorded as a function of the angle of polarization for fundamental light. (B) Monomer gold nanoparticle, (C) nano-antenna with 3 nm monomer separation, (D) nano-antenna with 22 nm monomer separation and (E) distance dependence normalized TPS intensity from nano-antenna with different separation distances. Here we have reported normalized TPS intensity (*I*nano-antenna2*ω*/*I*monomer2*ω*) with respect to monomer. Our distance dependence reported data clearly indicate that TPS spectroscopy ruler can be used within the 25 nm distance. FDTD simulations profile of distance dependent SH intensity from nano-antenna also has been included, which indicate that the theoretical fit matches very well with the experimental observation.

1 *I*_TPS_ = *GN*_w_*β*_w_^2^ + *N*_NP_*β*_NP_^2^*I*_*ω*_^2^e^[–*N*_NP_*l*(*α*_2*ω*_+2*α*_*ω*_)]^

where *G* is a geometric factor, *N*_w_ denote the number of water molecules and *N*_NP_ are the number gold nano-antenna per unit volume. On the other hand, *β*_w_ and *β*_NP_ are the first hyperpolarizabilities of a single water molecule and a single gold nano-antenna. Similarly, *α*_*ω*_ and *α*_2*ω*_ are the absorption cross section at *ω* and 2*ω*, respectively. *l* is the path length and *I*_*ω*_ is the intensity of fundamental 760 nm light. Fig. 1F shows the spectrum from gold nano-antenna recorded for an excitation laser wavelength at 760 nm in the absence of 3 nm bandwidth interference filter. Our experimental data as shown in Fig. 1F clearly show a strong TPS spectral peak at 380 nm and a much weaker and broader two-photon excited luminescence peak with *λ*_max_ = 480 nm. The reported result indicates that we can easily eliminate contribution from the two-photon fluorescence in the recorded TPS signal. As shown in the Fig. 2A, the two-photon scattering intensities from gold nano-antenna with 3 nm separation exhibit a linear relationship with the square of the powers of the fundamental incident light, which clearly implies that the signal is indeed due to the two-photon scattering process.

Our reported data clearly shows that TPS intensity is highly sensitive to small changes in the monomer separation distance and as a result, an optical ruler can be developed. Our results indicate that after a 30 nm separation, the TPS intensity from gold nano-antenna became the same as for a monomer gold nanoparticle. It is quite remarkable to see that TPS intensity ratio is greater than one for nano-antenna when the monomer is separated by 25 nm, a distance more than twice that of 10 nm for well documented FRET ruler.

Next, to understand the origin of high TPS intensity for nano-antenna with respect to the monomer gold nanoparticle, angle-resolved two-photon scattering measurement were performed using 760 nm incident light from a femtosecond Ti:sapphire laser. For angle-resolved TPS measurement, the fundamental beam at 760 nm was linearly polarized, whereas the input angle of polarization was selected using a rotating half-wave plate. It is now well documented that due to the symmetry consideration, two-photon scattering or the second-harmonic process is forbidden for cubic metals such as gold within the dipolar approximation.^22–30^ On the other hand, since for plasmonic gold nanoparticle systems, surfaces are known to play a major role, two-photon scattering arises from imperfect metallic nanostructure surfaces.^22–30,32–34^ Also in the case of nano-antenna, symmetry is broken at the interface. The two-photon scattering properties for metal nanoparticles that are small compared to the excitation wavelength of 760 nm (*d* ≪ *λ*/10) can be explained usually in the framework of electric-dipole approximation.^22–30,32–34^ As shown in Fig. 2B and D, our angle-resolved two-photon scattering measurement clearly shows that for the 25 nm monomer gold nanoparticle or nano-antenna with 22 nm separation, the two-photon scattering response is governed by the electric-dipolar contribution which arises due to the deviation of the particle shape from that of a perfect sphere. On the other hand, in case of nano-antenna with 3 nm monomer separation, since the size of the nano-antenna (25 nm + 3 nm + 25 nm) is comparable with one tenth of the wavelength of the incident light, contribution of higher-multipoles such as electric quadrupole becomes very important to the two-photon scattering spectra. To further characterize the nature of the coupled plasmon mode, a polarization analysis is performed by rotating the linear excitation polarization using a half-wave plate. In this experiment, two-photon scattering modes were selected to be polarized perpendicular to the scattering plane using an analyzer. Fig. 2C shows the polar plots for the two-photon scattering signal as a function of the angle of polarization. The observed asymmetry in the polarization line shape for the gold nano-antenna with 3 nm monomer separation, clearly evidences of the deficiency of the electric-dipole approximation for this system. The above data shows clear indication of the presence of higher multipolar interactions in these nanostructures, as a result, we have observed four lobes oriented on the 45, 135, 225 and 315° axes. Very strong two-photon scattering intensity from the gold nano-antenna reported here can be due to several factors and these are, (i) electric dipole contribution due to the imperfect structure of each nanoparticle, (ii) dipole contribution due to the broken symmetry at the interface. (iii) Electric quadrupole contribution due to the size effects, (iv) enhancement of electric dipole and electric quadrupole contribution due to the enhancement of local electric fields *via* strong plasmon coupling. Another important factor that may cause very strong TPS intensity from gold nano-antenna is single photon resonance.^33,34^ As reported in Table 2, our experimental data show that in gold nano-antenna with 3 nm separation, *λ*_PR_ is red shifted from 528 to 580 nm. As a result, single-photon resonance enhancement contribution in TPS intensity will be much higher for gold nano-antenna with shorter distance separation than corresponding monomer.

**Fig. 3:**
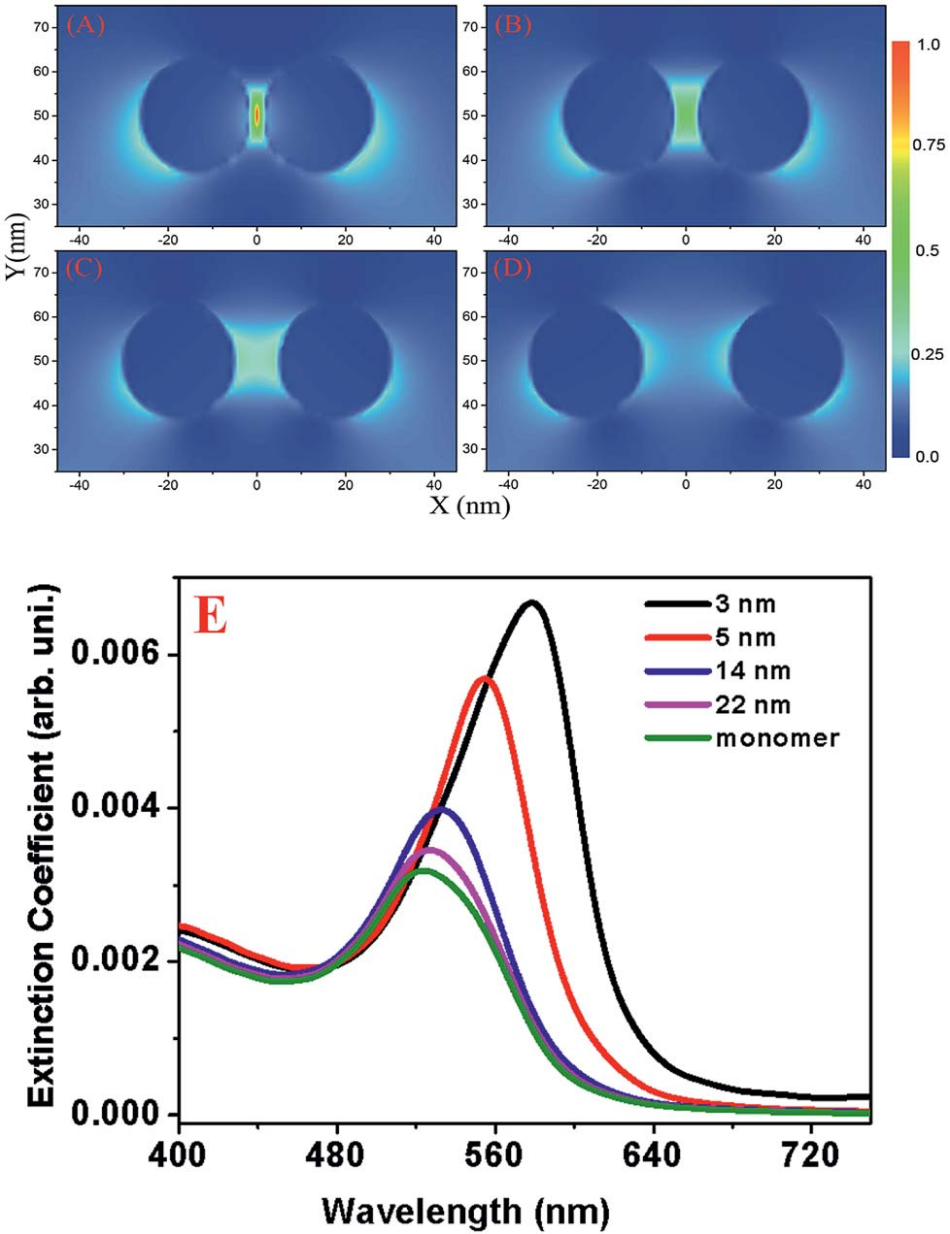
Near-field distribution of two-photon scattering or second-harmonic intensity for nano-antenna with different spacing: (A) 3 nm, (B) 5 nm, (C) 10 nm and (D) 22 nm. (E) Simulated extinction spectra for monomer and nano-antenna with different spacing.

To gain more insight into the effect of plasmon coupling on TPS intensity and ruler length, full-wave simulations were performed based on numerical solutions of Maxwell's equations, using the finite difference time domain (FDTD) simulation package.^21,24–26,30,32^ In these simulations, multipolar and finite size effects were included, which is very important for gold nano-antenna whose size is close to one tenth of the wavelength of fundamental excitation light. Recent reports shows that FDTD technique is suitable for plasmonic simulations and capable of finding electric *E*(*t*) and magnetic *H*(*t*) components of the electromagnetic coupling and also simulating the second harmonic intensity.^21,24–26,30,32^ Fig. 3 and Table 2 shows the simulated extinction spectra for nano-antenna with different spacing. From the theoretical study we found that plasmonic resonance occurs near 528 nm in the single nanoparticle, which matches quite well with experimental observation. In case of nano-antenna, plasmonic resonance closely relies on the gap distance, which also follows the reported experimental trend nicely. As reported in Fig. 3, our FDTD simulations results show the near field distribution of second harmonic intensity for nano-antenna with different spacing. Simulation results indicate a huge enhancement of TPS intensity for gold nano-antenna with respect to monomer gold nanoparticle, which matches quite well with experimental observation.

Both experimental and theoretical TPS intensity data, as shown in Fig. 2E also indicate huge TPS intensity enhancement for gold nano-antenna with 3 nm distance separation. As shown in Fig. 2E, our experimental finding on distance dependent two-photon scattering intensity for the “spectroscopy ruler” are well supported by FDTD simulation investigation. Both the theoretical and experimental data clearly shows that our “two-photon spectroscopy” ruler can be used within the 25 nm window.

To understand how the multipolar contribution changes with the separation distance between two gold nanoparticle monomers in gold nano-antenna, angle resolved two-photon scattering measurements were performed for gold nano-antennas with different separation distances. As shown in Fig. 2C, the polar plots clearly indicate that multipolar contribution is predominant for gold nano-antenna with 3 nm separation. Recently, Chandra *et al.* ^34^ have reported the influence of nanoparticle distance separation on SHG polarization and their reported result showed a similar plasmon ruler type of effect for shorter distance separation. Fig. 2D clearly shows that mainly dipolar contribution is predominant for gold nano-antenna with 22 nm monomer separation. All the above angle-resolved two-photon scattering measurement data and distance dependent TPS intensity data as reported in Fig. 2E, clearly indicate that the multipolar contribution plays a very important role for enormous TPS scattering intensity from gold nano-antenna.

**Fig. 4:**
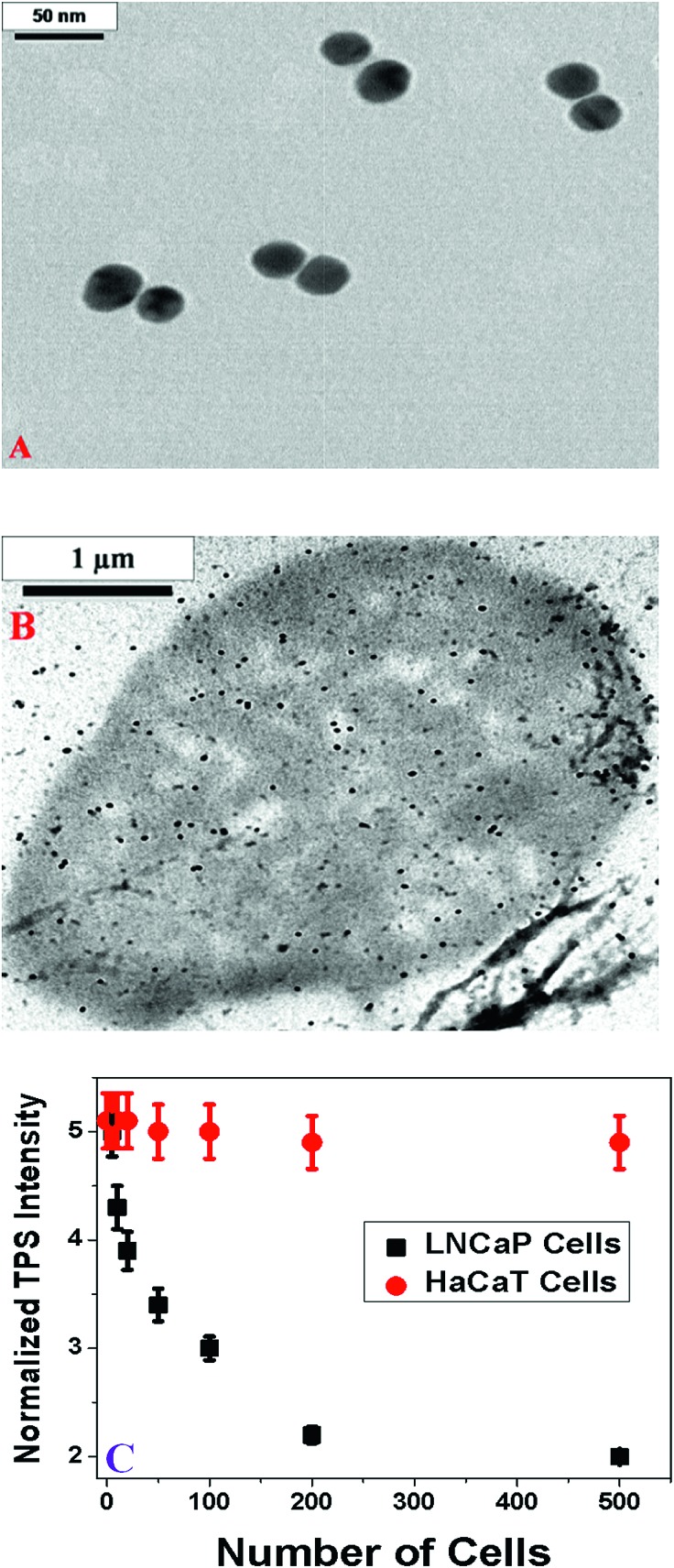
(A) TEM image shows TPS ruler made using partial sequence of A9 aptamer where two nanoparticles are separated by thiolated 12 bp ds-RNA. (B) TEM image shows A9 aptamer attached gold nanoparticles are bound on PSMA positive LNCaP cells, when 100 cancer cells were added. It also shows that due to the presence of PSMA in LNCaP cells, most of the dsRNA is dehybridized. (C) Plot shows how the normalized TPS intensity (*I*nano-antenna2*ω*/*I*monomer2*ω*) varies in the presence of different numbers of cells. Our result clearly shows that A9 aptamer based TPS ruler can be used for screening of PSMA (+) cancer cells selectively even at a concentration of 5 cells per mL.

Next to demonstrate possible application of TPS ruler for PSMA (+) cancer cell screening, we have developed spectroscopy ruler using partial sequence of the anti-PSMA A9 aptamer (5′-GGG AGG ACG AUG CGG ACC GAA AAA GAC CUG ACU UCU AUA CUA AGU CUA CGU UCC CAG ACG ACU CGC CCG–(CH_2_)_6_-3′–SH). The underlined sequence only has been used for hybridization purpose. For this purpose, HS–(CH_2_)_6_-modified A9 aptamer were attached with gold nanoparticles *via* the –SH group. Similarly HS–(CH_2_)_3_-modified capture oligonucleotide which was complementary to the underlined sequence of A9 aptamer was attached with different gold nanoparticles. After that, hybridization was performed in 10 mM PBS solution containing 0.3 M NaCl, for developing the TPS ruler, as shown Fig. 4A.

**Scheme 2:**
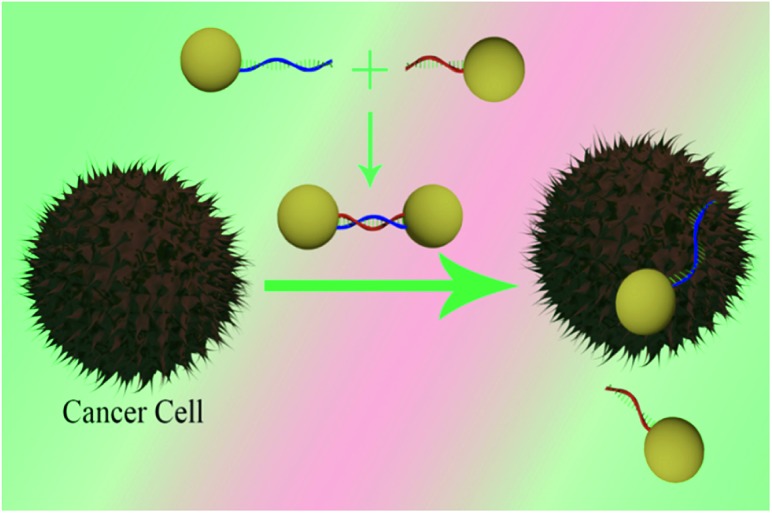
Schematic representation shows the development of the distance dependent two-photon scattering assay for screening of PSMA (+) cancer cell.

To demonstrate the PSMA (+) cancer cell screening capability, we used two different cell lines in the current experiment. The well-characterized LNCaP prostate cancer cell line, which over-expresses PSMA on the cell surface, was used.^35–37^ To understand the selectivity of cell screening process using TPS ruler, the PSMA (–) human skin HaCaT keratinocytes, a normal skin cell line was also used. Using an enzyme-linked immunosorbent assay kit, the measured amount of PSMA in LNCaP cells was 1.2 × 10^6^ per cell. No PSMA was found in HaCaT cells. Our TPS ruler based PSMA (+) cancer cell screening is based on the fact that when A9 aptamers based rulers are exposed to PSMA, a free portion of the aptamers will fold in such a way that it will specifically bind to the target PSMA on the cell surface. As a result, in the presence of PSMA (+) LNCaP cancer cells, nano-antenna are broken and will form monomers, as shown in Scheme 2 and Fig. 4B. Due to the above process, in the presence of PSMA (+) cancer cells, the spectroscopy ruler distance will change abruptly, which will decrease the two-photon scattering signal tremendously, as shown in Fig. 4C. In our study, we have monitored the normalized TPS intensity change to screen the PSAM (+) cells. Fig. 4C clearly shows that as the LnCaP cells concentration increases, the TPS intensity decreases. It is very interesting to note from Fig. 4C that the sensitivity of PSAM (+) LNCaP cell screening process is about 5 cells per mL.

Since the size of PSAM (+) LNCaP cells are around 10 μM and also the number of PSMA in a single cell is about 10^6^, one cancer cell can bind with huge amount of A9 aptamer attached gold nanoparticles, as a result, TPS intensity changes by about 9% even in the presence of 5 PSMA (+) LNCaP cells per mL. As shown in Fig. 4C, TPS intensity remains constant in the presence of PSAM (–) normal skin HaCaT cells, which clearly indicate that TPS ruler based PSAM (+) cells screening process is highly selective for cells which over-express PSMA on their cell surface.

## Conclusion

In conclusion, the current article reports for the first time, the development of rigid long range two-photon scattering nano-ruler whose optical window can be tuned within a 25 nm distance. Our design is based on the formation of gold nano-antenna, where monomer gold nanoparticles are separated by ds-DNA. Our experimental data indicate that the huge enhancement of two-photon scattering intensity is due to the formation of gold nano-antenna. Our reported data show that the two-photon scattering intensity from the nanoruler is highly sensitive to small changes in the monomer separation distance up to 25 nm. Experimental data with angle-resolved two-photon scattering measurement clearly shows that the multipolar contribution plays a very important role for very high TPS scattering intensity from gold nano-antenna. Theoretical insight using FDTD simulations supported our experimental observation very nicely. Simulated electric field |*E*|^2^ enhancement profile shows that the electric field enhancement decreases abruptly as the optical ruler distance increases. Though we have demonstrated that TPS ruler optical window can be tuned up to 25 nm distance, the distance limit can be varied by changing the size of spherical gold nanoparticle. Since the local electromagnetic field strengths vary with the particle size, it is expected that the distance limit will vary with the size of the nanoparticle. We have also demonstrated that a long range TPS ruler using A9 aptamer can be used for the screening of prostate-specific membrane antigen (PSMA) (+) prostate cancer cells, whose sensitivity is 5 cells per mL. Using PSMA (–) normal skin HaCaT cell, we have shown that TPS ruler based cancer cell screening assay is highly selective and it has capability to enable distinction from non-targeted cell lines. After appropriate engineering construction, long-range two-photon scattering rulers have the capability to be useful for better understanding of biological process.

## Materials and experiments

Sodium borohydride, sodium dodecyl sulfate (SDS), sodium chloride, buffer, trisodium citrate and hydrogen tetrachloroaurate were obtained from Sigma-Aldrich. ss-DNA or A9 aptamer strands with –SH modification were purchased from Midland Certified Reagent. PSMA (+) cancer cell lines and PSMA (–) cell lines and the growth media to grow cells, were purchased from the American Type Culture Collection (ATCC, Rockville, MD).

### Synthesis of gold nanoparticles

Gold nanoparticles of around 25 nm size were synthesized by using HAuCl_4_·3H_2_O and sodium citrate, using our reported procedure.^22,23,35^ A JEM-2100F transmission electron microscope (TEM), UV-visible absorption spectrum and Malvern Zetasizer Nano instrument were used for the characterization of nanoparticles.

### Synthesis of single-stranded DNA (ssDNA) attached gold nanoparticle

ssDNA (HS–(CH_2_)_6_–oligo) were attached to gold nanoparticle *via* thiol–gold chemistry. –SH modified single strand DNA with different lengths were gradually exposed to gold nanoparticles in the presence of sodium dodecyl sulfate, sodium chloride and PBS buffer for about 12–14 h. After attachment, unbound DNA strands were removed by centrifugation of the solution at 6000 rpm for 20 min. Similarly, complementary sequence (oligo–(CH_2_)_3_–SH) attached gold nanoparticle were prepared in 10 mM PBS solution containing 0.3 M sodium chloride. By using 5′-Rh6G modified ss-DNA strands and 10 μM potassium cyanide to oxidize the gold nanoparticles, about 80–90 DNAs per gold nanoparticle were estimated from fluorescence experiments.

### Design of two-photon spectroscopy rulers with different length

To design two-photon scattering spectroscopy rulers of different lengths, hybridization was performed using complementary sequence attached gold nanoparticles in 10 mM PBS solution containing 0.3 M NaCl. Excess ssDNA attached gold nanoparticles which were not involved in hybridization were removed by centrifugation from the solution. In our design, we used dsDNA spacers of 4, 10, 20, 30, 40, 60 and 80 base pairs to design a spectroscopy ruler window from 3 to 30 nm. The following capture sequences were used to design the spectroscopy rulers.

 

4mer CTG G-3′-SH.

10mer CTG GTC ATG G-3′-SH.

20mer CTG GTC ATG GCG GGC ATT TA-3′-SH.

30mer CTG GTC ATG GCG GGC ATT TAA TTC TCG GGC-3′-SH.

40mer CTG TTC GCG CTG GTC ATG GCG GGC ATT TAA TTC TCG GGC A-3′-SH.

60mer CTG TTC GCG CTG GTC ATG GCG GGC ATT TAA TTC TCG GGC ACG CCG TAG TTT GAA GTT ATC-3′-SH.

80mer CTG TTC GCG CTG GTC ATG GCG GGC ATT TAA TTC TCG GGC ACG CCG TAG TTT GAA GTT ATC CTG GTC ATG GCG GGC ATT TA-3′-SH.

### Two-photon scattering intensity measurements

The hyper-Rayleigh scattering technique was used to measure the two-photon scattering intensity as we and others have previously reported.^22,23,30^ For the measurement of TPS intensity, a fundamental wavelength of 760 nm from a mode-locked Ti:sapphire laser, having 150 fs pulse duration at a repetition rate of 80 MHz, was used. Detailed experimental procedures were previously reported. To make sure that there was no photo-thermal damage during TPS intensity collections, we performed TEM analysis before and after the exposure of laser, which indicates no photothermal damage from gold nano-antenna within our data collection time. To remove single photon scattering signals and signals from other nonlinear processes, we used a high-pass filter, 3 nm bandwidth interference filter, and a monochromator before the two-photon scattering light was detected by a cooled photomultiplier tube. For angle-resolved TPS measurement, the fundamental beam at 760 nm was linearly polarized, whereas the input angle of polarization was selected using a rotating half-wave plate.

### Finite difference time domain (FDTD) simulation

Full-field electromagnetic wave calculations were performed using an FDTD simulation software package.^21,24–26,30^ The discrete electric and the magnetic fields were evaluated on the grid points that are interspersed in both space and time. Frequency dependent dielectric function of gold was used for the gold nano-antenna. This method consists of discretization of electric field and magnetic field simultaneously in space and time. For our simulation, we used the experimental excitation wavelength for excitation of gold nano-antenna. Absorbing boundary condition was used for the entire calculations. The amplitude of the electric field was 1 V m^–1^. The courant number was taken as 0.99. The entire simulation is done with 0.001 nm mesh resolution and 4000 fs time for all cases.

### Cell culture

PSMA (+) cancer cells and normal cells were grown according to the ATCC procedure, as we have reported before.^30,36^ LNCaP cells were grown in a 5% CO_2_ incubator at 37 °C using ATCC medium supplemented with 10% premium fetal bovine serum (FBS) and antibiotics (10 IU mL^–1^ penicillin G and streptomycin) in 75 cm^2^ tissue culture flasks. The HaCaT cells were also grown in Dulbecco's Modified Eagle's Medicum (DMEM), as instructed by ATCC.
